# Non-oral manifestations in adults with a clinical and molecularly confirmed diagnosis of periodontal Ehlers-Danlos syndrome

**DOI:** 10.3389/fgene.2023.1136339

**Published:** 2023-05-31

**Authors:** C. Angwin, J. Zschocke, T. Kammin, E. Björck, J. Bowen, A. F. Brady, H. Burns, C. Cummings, R. Gardner, N. Ghali, R. Gröbner, J. Harris, M. Higgins, D. Johnson, U. Lepperdinger, D. Milnes, F. M. Pope, R. Sehra, I. Kapferer-Seebacher, G. Sobey, F. S. Van Dijk

**Affiliations:** ^1^ National EDS Service, London North West University Healthcare NHS Trust, London, United Kingdom; ^2^ Department of Metabolism, Digestion and Reproduction, Section of Genetics and Genomics, Imperial College London, London, United Kingdom; ^3^ Institute of Human Genetics, Medical University Innsbruck, Innsbruck, Austria; ^4^ National EDS Diagnostic Service, Sheffield Children’s NHS Foundation Trust, Sheffield, United Kingdom; ^5^ Clinical Genetics, Karolinska University Hospital, Solna, Sweden; ^6^ Department Otolaryngology Head and Neck Surgery, Children’s Health QLD, Brisbane, QLD, Australia; ^7^ School of Medicine, University of Queensland, Brisbane, QLD, Australia; ^8^ Clinical Genetics, Genetic Health Queensland, Brisbane, QLD, Australia; ^9^ Department of Operative and Restorative Dentistry, Medical University of Innsbruck, Innsbruck, Austria; ^10^ Department of Dermatology, Chelsea and Westminster Hospital NHS Foundation Trust, London, United Kingdom

**Keywords:** periodontitis, Ehlers-Danlos syndrome, complement, non-oral, genetics

## Abstract

**Introduction:** Periodontal Ehlers-Danlos Syndrome (pEDS) is a rare autosomal dominant type of EDS characterised by severe early-onset periodontitis, lack of attached gingiva, pretibial plaques, joint hypermobility and skin hyperextensibility as per the 2017 International EDS Classification. In 2016, deleterious pathogenic heterozygous variants were identified in *C1R* and *C1S*, which encode components of the complement system.

**Materials and Methods:** Individuals with a clinical suspicion of pEDS were clinically and molecularly assessed through the National EDS Service in London and Sheffield and in genetic services in Austria, Sweden and Australia. Transmission electron microscopy and fibroblast studies were performed in a small subset of patients.

**Results:** A total of 21 adults from 12 families were clinically and molecularly diagnosed with pEDS, with *C1R* variants in all families. The age at molecular diagnosis ranged from 21–73 years (mean 45 years), male: female ratio 5:16. Features of easy bruising (90%), pretibial plaques (81%), skin fragility (71%), joint hypermobility (24%) and vocal changes (38%) were identified as well as leukodystrophy in 89% of those imaged.

**Discussion:** This cohort highlights the clinical features of pEDS in adults and contributes several important additional clinical features as well as novel deleterious variants to current knowledge. Hypothetical pathogenic mechanisms which may help to progress understanding and management of pEDS are also discussed.

## Introduction

Ehlers-Danlos Syndromes (EDS) are a heterogeneous group of rare monogenic conditions that are characterized by joint hypermobility, skin and vascular fragility and generalised connective tissue friability (Malfait, 2018). Currently, there are 14 types recognized, 13 with monogenic causes by variants in 20 different genes, the majority of which encode fibrillary collagen types I, III, and V, modifying or processing enzymes (for example, collagenases and lysyl hydroxylases) or those proteins and enzymes that modify the extracellular matrix (for example, tenascin-X) (Malfait et al., 2020).

Periodontal EDS (pEDS) was first described in 1977 (Stewart et al., 1977). Currently, pEDS is diagnosed by the following major criteria: (i) severe early onset periodontitis, (ii) lack of attached gingiva, (iii) pretibial plaques, and (iv) family history of an affected first degree relative, and minor criteria: (i) easy bruising, (ii) distal joint hypermobility, (iii) skin hyperextensibility/fragility/wide or atrophic scarring, (iv) increased infection rate, (v) hernias, (vi) marfanoid facial features, (vii) acrogeria, and (viii) prominent vasculature (Malfait et al., 2017). A clinical diagnosis of pEDS can be made through a combination of criteria, the presence of major criterion (i) or major criterion (ii), plus at least two other major criteria and one minor criterion (Malfait et al., 2017).

Since the first description, pEDS has been described clinically in 165 individuals in several case reports, series and pedigree analyses (Nelson and King, 1981; Biesecker et al., 1991; Rahman et al., 2003; Reinstein et al., 2011; Reinstein et al., 2012; Reinstein et al., 2013; Kapferer-Seebacher et al., 2016; Kapferer-Seebacher et al., 2017; Cortés-Bretón Brinkmann et al., 2021; El Chehadeh et al., 2021; Stock et al., 2021; Lepperdinger et al., 2022; Nakajima et al., 2022). In 2003, linkage studies in 5 pedigrees with clinical features of pEDS identified a locus at 12p13 (Rahman et al., 2003). However, it was not until 2016 that heterozygous pathogenic variants in the genes *C1R* (MIM 613785, HGNC 1246) and *C1S* (MIM 120580, HGNC 1247) were found to be causative of pEDS (Kapferer-Seebacher et al., 2016). Unlike causative variants in other rare types, these genes do not encode proteins involved in collagen I, III, or V biosynthesis or modification of proteoglycans. Instead, they encode the protein esterases C1r and C1s, subunits of the complement 1 complex. Activation of C1r and C1s is the first step in the classical complement cascade, a major antimicrobial pathway of the innate immune system. The pathogenesis of pEDS is only partly understood; evidence suggests that oral features are linked to secretion or release of active C1r serine protease in the extracellular space. This mechanism may cause gingival hyperinflammation in response to mild biofilm accumulation, and subsequently rapidly progressing periodontal destruction leading to dental loss. (Kapferer-Seebacher et al., 2016; Kapferer-Seebacher et al., 2020). The question arises whether this hyperinflammation is also the mechanism of several non-oral features observed in pEDS (Kapferer-Seebacher et al., 2016; Kapferer-Seebacher et al., 2020).

In the current literature, 165 individuals from 34 families have been published, with 27 (likely) pathogenic variants (*C1S* = 5 variants, *C1R* = 22 variants) (Kapferer-Seebacher et al., 2016; Wu et al., 2018; Kapferer-Seebacher et al., 2019; Kapferer-Seebacher et al., 2020; Cortés-Bretón Brinkmann et al., 2021; El Chehadeh et al., 2021; Stock et al., 2021; Lepperdinger et al., 2022; Nakajima et al., 2022). Although the molecular cause for pEDS has been defined and fundamental studies are being undertaken to elucidate the pathogenic mechanism, (Kapferer-Seebacher et al., 2016), there is also a need for detailed phenotyping of molecularly confirmed adults with pEDS, to identify specific associated clinical features to improve diagnosis, understanding of pathogenesis and management. Of importance, other clinical features have recently been reported in pEDS including leukodystrophy (Kapferer-Seebacher et al., 2019) and hoarseness of voice (George et al., 2016). A recent paper reported a cohort of molecularly diagnosed individuals with pEDS and vascular abnormalities including venous insufficiency and arterial aneurysms (El Chehadeh et al., 2021). Here, we report on a spectrum of non-oral features in 21 adult individuals from 12 families with a clinically and molecularly confirmed diagnosis of pEDS with the aim of developing our understanding of clinical features and underlying pathogenic mechanisms.

## Materials and methods

### Patients

Patients with a suspicion of pEDS were seen in the National EDS Service in London and Sheffield and in genetic centres in Austria, Sweden and Australia. Patients with a confirmed clinical and molecular diagnosis of pEDS were included in the study. The patients were reviewed over a study period of 2019–2023. Photographs of facial features were assessed independently by three consultant geneticists, if not possible, descriptions of facial features were taken from the notes. Written consent for publication, including photographs, was obtained from all individuals. According to the Institutional Review Board (IRB) no formal research ethics approval or research and development approval was required as stipulated by the United Kingdom Policy Framework for Health and Social Care Research and the Health Research Authority decision tool.

### Transmission electron microscopy and collagen electrophoresis

As part of the diagnostic process a subset of patients underwent a skin biopsy for transmission electron microscopy (TEM) (Angwin et al., 2020) and collagen electrophoresis with methodology as described by (Körkkö et al., 1998). The majority of biopsies were taken from the inner, upper forearm in order to maintain consistency of samples across the cohort.

### Molecular analysis

Molecular analysis was carried out via massively-parallel sequencing (NextSeq, Illumina), and data analysis using SeqNext software and CNV Detective. Variants were analysed according to best practice guidelines for the evaluation of pathogenicity and the reporting of sequence variants in clinical molecular genetics (Richards et al., 2015). Confirmation of clinically significant sequence variants by Sanger sequencing was performed as necessary. DNA changes have been described according to NM_001733.4 for *C1R* and NM_201442.3 for *C1S* and validated via Variant Validator (Freeman et al., 2018).

## Results

### Patients

A total of 21 adults (P1-21) from 12 families were diagnosed with pEDS, see Table 1; data on children with a confirmed molecular diagnosis of pEDS were reported previously (Kapferer-Seebacher et al., 2020). Individuals P4, P5, P9 were reported without clinical details (members of families C, E, and B, respectively) in a study by Rahman et al. demonstrating linkage to locus 12p13 (Rahman et al., 2003) and have been confirmed to have a diagnosis of pEDS in the present study. Only P13 has been clinically and molecularly reported before (George et al., 2016; Kapferer-Seebacher et al., 2016). Individuals were seen in the national EDS services in London (*n* = 13) and Sheffield (*n* = 5) and in the genetic services in Austria (*n* = 2) and Sweden (*n* = 1). The age at molecular diagnosis ranged from 21–75 years (mean 44 years). Two siblings passed away at the ages of 30 and 41, and have not been included in age of diagnosis as testing took place after death. The male: female ratio was 5:16. Ethnic backgrounds were White British, Swedish, Austrian and Latvian. Please see Supplementary Table S1 for clinical features. Four additional individuals from two families have been included in the Supplementary appendix, as they have a clinical diagnosis of pEDS but there is uncertainty about the pathogenicity of the identified variant.

**TABLE 1:** Cohort demographics and clinical features ‘+’ = sign is present, ‘-‘ = sign is absent, U = data unknown.

Individual	P1	P2	P3	P4	P5	P6	P7	P8	P9	P10	P11	P12	P13	P14	P15	P16	P17	P18	P19	P20	P21	Total
Gender	F	M	F	F	F	M	F	F	F	M	F	F	F	F	F	F	F	M	F	M	F	M = 5 (24%)
																						F = 16 (76%)
Age at molecular diagnosis	33	69	73	69	60	21	40	45	59	34	41	30	39	45	24	75	28	35	33	29	26	
Gene	*C1R*	*C1R*	*C1R*	*C1R*	*C1R*	*C1R*	*C1R*	*C1R*	*C1R*	*C1R*	*C1R*	na	*C1R*	*C1R*	*C1R*	*C1R*	*C1R*	*C1R*	*C1R*	*C1R*	*C1R*	*C1R* = 20 (95%
Variant	c. (1273 + 1_1274-1)_(1348 + 1_1349-1)del	c.707T>C	c.689T>C	c.689T>C	c. (1273 + 1_1274-1)_(1348 + 1_1349-1)del	c.1073G>A	c.689T>C	c.689T>C	c.702_711delinsT	c.702_711delinsT	c.926G>A	na	c.869A>G	c.149_150 TC>AT	c.149_150 TC>AT	c.1339T>C	c.628T>G	c.1113C>G	c.1113C>G	c.1073G>T	c.1073G>T	
Protein	exon 10 deletion	p. (Phe236Ser)	p. (Leu230Pro)	p. (Leu230Pro)	exon 10 deletion	p. (Cys358Tyr)	p. (Leu230Pro)	p. (Leu230Pro)	p. (Lys235_Leu237del)	p. (Lys235_Leu237del)	p. (Cys309Tyr)	na	p. (Asp290Gly)	p. (Val50Asp)	p. (Val50Asp)	p. (Cys447Arg)	p. (Tyr210Asp)	p. (Cys371Trp)	p. (Cys371Trp)	p. (Cys358Phe)	p. (Cys358Phe)	
Dental																						
Periodonititis	+	+	+	+	+	+	+	+	U	+	U	U	+	+	+	+	+	+	+	+	+	18 (86%)
Lack of attached gingiva	+	+	+	+	+	+	+	+	U	+	U	U	+	+	+	+	+	+	+	+	+	18 (86%)
Dermatological features																						
Thin skin	+	+	+	+	+	+	-	-	U	-	+	U	+	-	-	+	-	+	+	+	+	13 (62%)
Hyperextensibiliy	-	-	-	-	-	-	-	-	+	-	-	U	-	-	-	U	-	+	-	+	+	4 (19%)
Fragility	-	+	+	+	+	+	+	+	+	+	U	U	+	-	-	+	-	+	+	+	+	15 (71%)
Easy bruising	+	+	+	+	+	+	+	+	+	+	+	U	+	+	+	+	-	+	+	+	+	19 (90%)
Long term bruising	-	+	+	+	+	+	U	+	U	+	U	U	-	-	-	U	-	+	+	+	+	11 (52%)
Pretibial discolouration	-	+	+	+	+	+	+	+	+	+	+	+	+	-	-	+	-	+	+	+	+	17 (81%)
Symmetrical discolouration	-	+	+	+	+	+	+	+ plus pigment on right calf	+	+	+	+	+ plus pigment on right calf	-	-	+	-	+	+	+	+	17 (81%)
Abnormal scarring	+ atrophic	-	+ widened	+ atrophic	-	+ atrophic	-	-	U	+ widened	-	U	+ atrophic	-	-	+ widened	-	-	+ atrophic	-	+ atrophic	9 (43%)
Thread veins	+	+	+	+	-	-	-	-	+	-	U	U	-	-	-	-	-	-	+	-	-	6 (29%)
Musculoskeletal																						
Beighton score	2/9	0/8	0/9	0/8	1/9	7/9	4/9	0/8	5/9	0/8	U	U	2/9	1/9	1/9	0/9	6/9	0/9	3/9	8/9	9/9	5 (24%)
Joint pain, age (yrs)	+	-	+	-	+	-	+	+	+	+	U	U	+	-	-	-	+	+	-	+	-	11 (52%)
	10						40	10	20	18							18	33		29		
Dislocation/subluxations	-	+	-	-	-	-	+	-	-	U	U	U	-	-	-	+	-	+	-	-	-	4 (19%)
Spinal abnormalities	+	-	-	+	U	+	-	-	U	-	U	U	-	-	-	-	-	-	-	-	-	3 (14%)
Foot abnormality	-	-	+	+	-	U	+	-	+	+	U	U	-	-	-	+	+	-	+	-	-	8 (38%)
Ear, nose and throat																						
Voice changes (age of onset in years)	-	+55	-	-	-	+19	-	-	-	-	U	U	+20	+ U	-	+ U	-	+19	-	+14	+ U	8 (38%)
Management		tracheal resection											surgical correction	antibiotics				speech and language therapy			Surgical correction	
Complications		mid anastomotic stricture treated via laser bronchoscopy																			Surgical resection of scar tissue with local steroid injections	
Central nervous system																						
Leukodystrophy (age at scan in years)	U	U	U	+64	U	U	+41	+46	U	U	U	+30	U	U	U	U	- 30	+38	+36	+33	+30	8/9 (89%)
Psychological symptoms	+	-	+	-	+	+	+	+	-	+	-	-	-	-	-	-	-	+	+	+	-	10 (48%)
Tremor	-	+	-	-	-	-	+	-	-	U	-	-	-	-	-	-	-	+	-	-	-	3 (14%)
Loss of concentration	-	-	-	-	U	-	+	-	+	U	U	U	-	-	-	-	-	+	-	+	+	5 (24%)
Diplopia	-	-	-	+	-	-	-	-	-	-	U	U	-	-	-	-	-	-	-	-	-	1 (5%)
Loss of speech	-	-	-	-	-	-	-	-	-	-	U	U	-	-	-	-	-	-	-	-	-	0
Spasticity	-	-	-	-	-	-	-	-	-	-	U	U	-	-	-	-	-	-	-	-	-	0
Memory loss	-	-	-	-	-	U	-	+	+	U	U	U	+	-	-	-	-	-	-	+	-	4 (19%)
Headache	+	-	-	+	-	U	+	+	U	U	U	U	-	-	-	+	-	+	+	+	+	9 (43%)
Migraine	-	-	-	-	-	-	+	+	U	U	U	U	-	-	-	+	-	+	-	+	+	6 (29%)
Incontinence	+	-	-	-	-	-	+	-	-	U	U	U	U	-	-	U	-	-	-	-	-	2 (10%)
Back pain	+	-	-	-	+	-	+	+	+	+	U	U	U	-	-	+	-	+	-	+	-	9 (43%)

### Transmission electron microscopy and collagen electrophoresis

TEM was carried out in 6/21 individuals. No consistent features were observed: Irregular collagen packing (P2, *n* = 1), variability in fibril diameter (P9, P21, *n* = 2), single collagen flower (P21, *n* = 1) and protein filled rough endoplasmic reticulum in fibroblasts (*n* = 2). In 1 individual, a biopsy was taken from an area of pretibial discoloration in early adulthood, and electron microscopy showed scarring with haemosiderin deposition, inflammatory changes with mainly perivascular nodular aggregates of lymphocytes, with fragmentation leading to clumping of elastin. Fibroblast cultures with protein studies were carried out in 7 patients; of these 6 (86%) had normal type III collagen and 1 (14%) had slightly increased production of type III collagen.

### Molecular analysis

**FIGURE 1:**
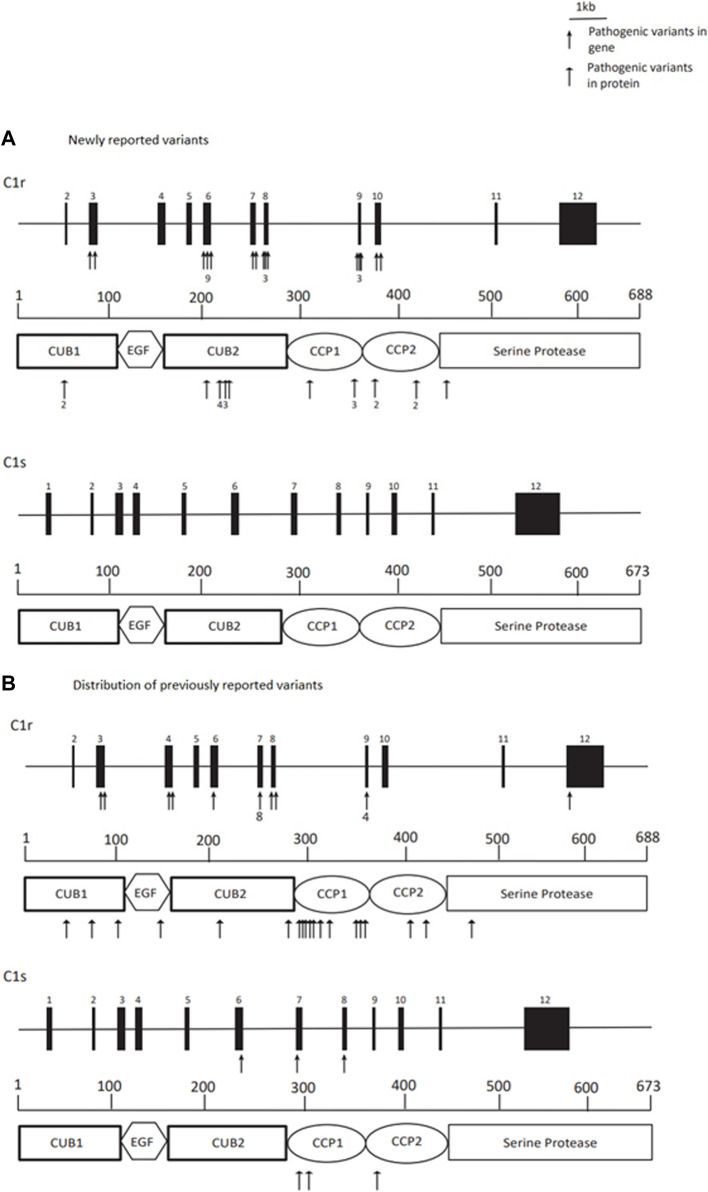
Title: Distribution of *C1R* (likely) pathogenic variants and their localisation in the C1r protein in newly reported variants from this paper, and previously reported variants in *C1R* and *C1S*. Legend. **(A)** Newly reported variants–deleterious variants are distributed throughout the *C1R* gene, however, there appears to be a cluster of alterations affecting the interaction and catalytic domains (CUB2 and CCP1/2) of the C1r protein. Arrows indicate reported variants, these are numbered where there are multiple. **(B)** Distribution of previously reported variants with clustering affecting the interaction and catalytic domains (CUB2 and CCP1/2) of both C1r and C1s proteins. Arrows indicate reported variants, these are numbered where there are multiple.

All identified deleterious variants are detailed in Supplementary Table S1. Identified variants were found in *C1R* in 12/12 (100%) families. In P11 the diagnosis was made on stored DNA after death. Her sibling (P12) with a comparable phenotype was no longer alive and was not tested. Six newly described variants included in this paper are as follows: (1) *C1R* c.628T>G, p.(Tyr210Asp), (2) *C1R* c.707T>C, p.(Phe236Ser), (3) *C1R* c.926G>A, p.(Cys309Tyr), (4) *C1R* c.1273 + 1_1274-1)_(1348 + 1_1349-1)del (exon 10 deletion), (5) *C1R* c.1273 + 1_1274-1,1348 + 1_1349-1del p.(Gly425_Pro449del), and (6) *C1R* c.1339T>C, p.(Cys447Arg). All the (likely) pathogenic variants identified in this cohort are compatible with the production of stable enzymatically active C1r protein, in line with the proposed activating effect (Gröbner et al., 2019). Variants are distributed throughout the *C1R* gene, however, there appears to be a cluster of pathogenic alterations affecting the C1r interaction and catalytic domains (CUB2 and CCP1/2) (Figure 1) (Gröbner et al., 2019).

## Discussion

This paper provides the first complete detailed phenotypic overview of non-oral features in a cohort of 21 adult individuals with a molecularly confirmed diagnosis of pEDS. Please see Supplementary Table S1 for detailed clinical features.

### Oral manifestations

Oral manifestations of the present cohort are reported in detail elsewhere (Lepperdinger et al., 2022).

#### Gingival recession

Thin and fragile gingiva and periodontitis lead to gingival recession that characterizes the oral image of people affected by pEDS. In the present cohort more than 90% of dentate individuals presented with gingival recession ≥3 mm. Gingival recession provides a useful and easily assessable oral characteristic in the diagnosis of pEDS and may give initial examiners the opportunity to easily support the suspected diagnosis of pEDS.

#### Early and severe periodontitis

All but two dentate (with retained teeth) individuals were diagnosed with severe periodontitis at young age (≤30 years). Age of first tooth loss due to periodontal reasons was reported to be at a median of 20 years. The probability of being edentate at age 35-44 years was 28%–47%. Two individuals with excellent oral hygiene and receiving professional tooth cleaning on a regular basis had no or only mild periodontal destruction. They were clinically diagnosed with pEDS based on a lack of attached gingiva, pretibial hemosiderin depositions and an affected first degree relative.

#### Lack of attached gingiva

**FIGURE 2:**
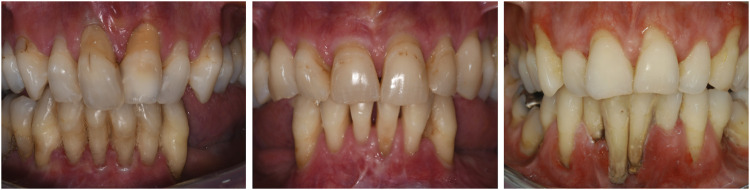
Title: Oral manifestations of pEDS. Legend: Thin and fragile gums (due to lack of attached gingiva) and periodontitis lead to gingival recession that characterizes the oral image of people affected by pEDS. Age of first tooth loss due to periodontal reasons was reported to be at a median of 20 years.

The oral feature observed in all dentate individuals who had a periodontal assessment was the generalized lack of attached gingiva (*n* = 18) (Figure 2). Unfortunately, three individuals (two deceased), did not have a complete periodontal examination. Generalized lack of attached gingiva has been reported to be pathognomonic for pEDS, and has been reported in children with pEDS in children as young as 4 years old (Kapferer-Seebacher et al., 2020).

### Skin

#### Pretibial plaques

Pretibial plaques are strongly associated with pEDS (reported frequency of 83% in the literature) (Kapferer-Seebacher et al., 2016) and occur in a specific pattern with hyperpigmentation, atrophy or induration and scarring of the lower limbs typically over the pretibial area, termed pretibial plaques (Kapferer-Seebacher et al., 2016; Malfait et al., 2020). Hyperpigmentation appears to develop gradually from mild, brownish hyperpigmented patches clinically in keeping with haemosiderin deposition, to indurated or atrophic plaques and can progress to cover the entire circumference of the calf. Plaques are often associated with skin fragility, whether atrophic or indurated.

**FIGURE 3:**
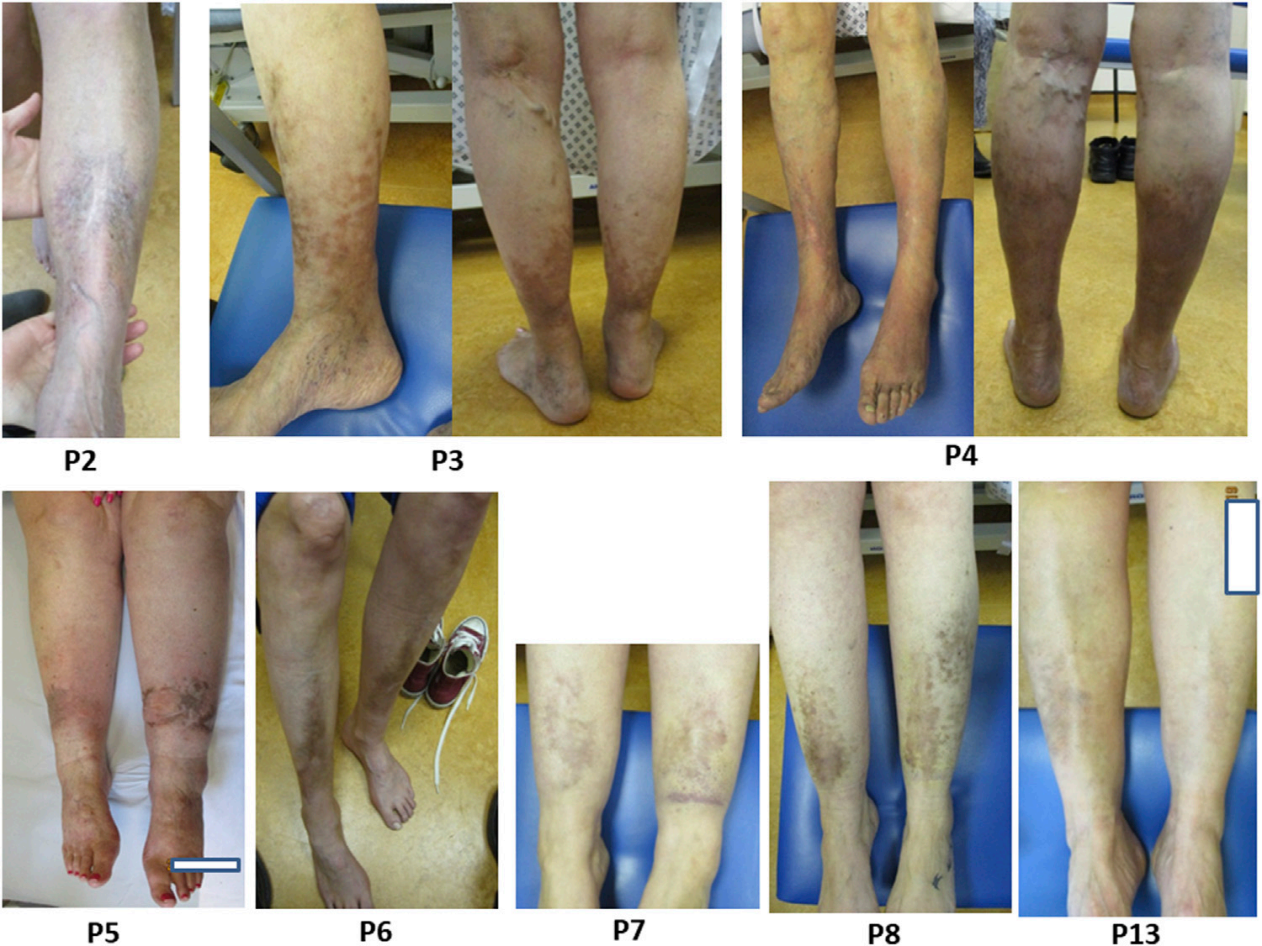
Title. Pretibial plaques in individuals with pEDS. Legend. Examples of pretibial plaques with symmetrical distribution and with some extending around the entire circumference of the calf, as observed in individuals P2, P3, P4, P5, P6, P7, P8 and P13.

In our cohort 17/21 (81%) had pretibial plaques, comparable to the literature (83%) (Figure 3). 14/17 had symmetrical pigment deposition. Plaques extended around the entire circumference of the calf in 5/17 (29%). We do not have data for age of onset, however in a recent cohort of affected children, only 2/12 (16%) reported pretibial plaques (Kapferer-Seebacher et al., 2020). Varicose veins had been diagnosed in 8/21 (38%); all these individuals also had pretibial hyperpigmentation of the lower limb, and 3 of these had extensive discolouration around the entire calf circumference. In the 4 (19%) individuals without pretibial plaques there were no common characteristics (age, underlying genetic cause, etc.) and one individual had a family member who was affected with pretibial plaques.

The mechanism for pretibial plaque development remains unclear. The discolouration may represent post-inflammatory hyperpigmentation after injury or inflammatory disorder of the skin. However, the symmetrical distribution seen in 14/17 (82%) individuals and level of hyperpigmentation is not in keeping with a purely trauma related process post-injury as is seen in other rare EDS types such as classical EDS (Bowen et al., 2017). Some individuals do not recall significant trauma to their lower limbs in the pattern of discolouration. The pretibial and calf area is a common site to be affected in disorders of metabolism or circulation, and this is, for example, seen in chronic venous disease, pretibial myxoedema in thyroid disease, necrobiosis lipoidica, pyoderma gangrenosum, and others. The proposed mechanism for this localisation is venous/lymphatic pooling of metabolites and immune complexes, resulting in increased local activity in comparison to the rest of the body (Fatourechi, 2005; Caggiati et al., 2008; Caggiati et al., 2010). A potential mechanism for the occurrence of pretibial plaques could be pooling of C1 components and related immune complexes in the lower limbs, resulting in a localised dermal inflammatory response. A recent study describing clinical features in children with pEDS who typically have no or mild cutaneous signs, including children of some of the adults included in this paper (family B, C, D, F correspond to Family 3, 5, 8, 12 respectively) (Kapferer-Seebacher et al., 2020). This could be in keeping with the hypothesis that the observed cutaneous changes are the result of chronic exposure to a higher concentration of overactivated complement components, resulting in inflammatory changes with associated hyperpigmentation and haemosiderin deposition.

It is possible that the majority of hyperpigmentary changes to the pretibial area in pEDS are a result of localized chronic inflammation, leading to melanosis, haemosiderin deposition, extracellular matrix (ECM) remodelling and angiogenesis. Further investigations are important to confirm or reject this hypothesis, for example, *in vitro* skin models, biopsy of skin and underlying tissue, and non-invasive investigations, e.g., ultrasound of microvasculature in a similar technique to (Ritelli et al., 2019).

Prolonged skin inflammation is a risk factor for development of cutaneous squamous cell carcinomas (cSCC) (Riihilä et al., 2019). Tumour derived C1r and C1s are thought to aid cSCC progression and have been suggested as an cSCC biomarker (Riihilä et al., 2020). In this cohort, P18 had an SCC excised from the area of pretibial discolouration with closure requiring skin grafting. An individual reported in the appendix has a history of multiple Basal Cell Carcinomas prior to her clinical diagnosis with pEDS (see Supplementary Appendix). Given the size of this cohort, there is no evidence of an association between increased rates of cutaneous cancers and pEDS. As with any individual, new masses on the skin in those affected by pEDS should be assessed and investigated thoroughly.

#### Easy bruising

Easy bruising was noted in 90% of individuals (19/21) compared to 95% in the literature. Easy bruising occurred particularly over the shins in 8/21 (38%). In 10 individuals bruising would take a prolonged period of time to fade, up to 9 months in one individual. A particular pattern of redness, swelling, pain and then prolonged bruising was observed in 2 (P8, P13) individuals after mild trauma; in P13 localised to the lower limbs and in P8 could affect any area of the body. Bruising related bleeding could contribute to haemosiderin deposition and hyperpigmentation, however one individual (P1, age 33) reported easy bruising with no evidence of pretibial changes.

Vitamin C use has been recommended as a method to possibly reduce bruising in different types of EDS. It is known to be involved and potentially increase collagen production by fibroblasts (Tajima and Pinnell, 1996; Bowen et al., 2017), however there is no current clinical evidence that it reduces bruising frequency or severity. Given that the pathophysiological mechanism of pEDS remains unclear, the role for this supplement in pEDS related bruising is unknown.

#### Skin hyperextensibility/fragility/wide or atrophic scarring

Hyperextensible skin was noted in 4 of 21 (19%) individuals. Fragility of the skin was found in 15/21 (71%), which was particularly concentrated over the legs and shins (*n* = 9). Skin graft was required for 1 individual after minor trauma to the shin. Abnormal scarring was reported in 10/21 (48%) and particularly prominent on the lower legs in 9 (although some also had abnormal scarring on arms, torso and head), described as: atrophic (*n* = 6), widened (*n* = 4) and keloid (*n* = 1).

#### Prominent vasculature and acrogeria

Thin, translucent skin was noted in 13/21 (62%) individuals within our cohort and specifically over the chest in 5 individuals. Acrogeria was not specifically noted.

### Musculoskeletal

In this cohort, distal hypermobility was seen in 5/19 (26%) individuals with 2 of those having a Beighton score of 5 or over. Three additional individuals had a Beighton score of 5 or over. Dislocations were reported by 2/21 (10%) individuals following appropriate trauma (fall down stairs and road traffic accident both resulting in shoulder dislocations). Joint pains were reported by 11/21 (52%) individuals (generalised in 5). Joint pains typically started in early adulthood (average age 30, age range of onset 10–60 years).

### Gastrointestinal

#### Hernia

Hernia was noted in 5/21 (23.8%) individuals: inguinal (*n* = 2), incisional (*n* = 1), umbilical (*n* = 1) and a combination of hiatus, inguinal and umbilical (*n* = 1).

#### Diverticular disease and bowel perforation

Diverticular disease was reported in 5/21 (24%) individuals, 1 of whom (P3) suffered a related bowel perforation. Generally, diverticulitis is considered a chronic inflammatory state, in conjunction with other lifestyle, genetic and environmental factors, at an incidence of 188/100,000 (Bharucha et al., 2015; Strate and Morris, 2019). Given its relatively high frequency in the population and the small cohort published here, no definite conclusions on association can be drawn.

Fatal spontaneous bowel perforation caused the death of individual P11 at age 41 and was also the cause of death in a sibling of P9 who passed away prior to genetic testing or assessment but had clinical features of pEDS. Organ rupture has been previously reported in 2 patients with molecularly confirmed pEDS, although the sites of perforation are not specified (Kapferer-Seebacher et al., 2016; Kapferer-Seebacher et al., 2017). Of note, the proband of family 1 with a clinical diagnosis of pEDS (see Supplementary Appendix) had two small bowel ruptures requiring bowel resections.

### Concurrent disorders

#### Recurrent infections

Recurrent infection has been included in the current diagnostic criteria (Malfait et al., 2017). In this cohort 7/21 (33%) individuals subjectively felt that they were more prone to recurrent infections (see Supplementary Table S1).

#### Inflammatory disorders

From medical history no increased infection rate was apparent. Complex Systemic Lupus Erythematosus (SLE) was reported in P10 (*C1R* (c.702_711delinsT)), with initial presentation of rash, joint aches and chest pain, developing a severe pericarditis requiring surgical intervention and a prolonged and resistant serositis. Multiple treatments for his SLE included Methotrexate, Hydroxychloroquine, Mepacrine, Rituximab and Azathioprine, however due to intolerance or minimal response he has continued to require long term steroids. P10 has recently developed three lesions on the left lower limb in keeping with a diagnosis of pyoderma gangrenosum, which have been minimally responsive to 60 mg of oral prednisolone and regular dressings. Infliximab is being considered as a management option. SLE had also been diagnosed in both siblings (did not participate in this study) of the individuals’ mother (P9). P9 was not affected with SLE. Unfortunately, these family members were not available for genetic testing.

Interestingly, a study of a consanguineous family with SLE identified recessive loss of function (LOF) variants in *C1R* (with low serum levels of complement) in contrast to dominant gain of function variants seen in pEDS (Demirkaya et al., 2017). Given the United Kingdom incidence of SLE is estimated at 4.91/100,000 in the general population, (Stojan and Petri, 2018), it is currently unclear whether there is an association between pEDS and SLE; this cohort is not large enough to draw any definite conclusions.

P5 has a diagnosis of palmoplantar pustular psoriasis (PPP). This is an inflammatory disease characterized by sterile neutrophilic pustules surrounded by inflamed or reddened/discoloured skin. The mechanism of disease is unknown but which appears to be mediated by aspects of both innate and acquired immune systems (Brunasso and Massone, 2021).

P20 was diagnosed at the age of 12 with mesangiocapillary glomerulonephritis type I (now known as C3 glomerulonephritis). This condition is part of the disease entity C3 glomerulopathy caused by dysregulation of the alternative complement pathway (rather than the classical pathway which is initiated by C1) (Sethi et al., 2012; Smith et al., 2019).

In summary, in 3/21 (14%) individuals an autoimmune condition was diagnosed with one individual having a diagnosis of SLE as well as two second-degree maternal family members who also had clinical features of pEDS (not included in this study). Autoimmune condition incidence varies by disorder; however, they are largely uncommon in the general population (Wang et al., 2015). Within this group there does appear to be a propensity toward autoimmune conditions, particularly those possibly due to complement dysregulation, however the cohort is not large enough to draw any definite conclusions.

### Facial features

**FIGURE 4:**
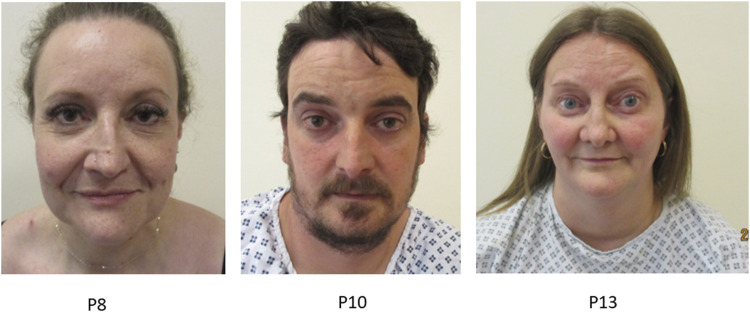
Title: Facial features in individuals with pEDS. Legend. Individuals with pEDS share facial features of that are more often seen in individuals with vascular EDS including prominent eyes, narrow nose and thin vermillion of the lips seen in individual P8, P10 and P13.

Although Marfanoid facial features are included as minor diagnostic criteria for pEDS, none of our series showed such changes. Contrastingly we consider that 6/21 (28.5%) had features resembling those of vEDS (vascular EDS). Facial features noted in vEDS are thin vermilion of the lips, micrognathia, narrow nose, and prominent eyes (Brady et al., 2017). Typical facial features in the cohort were thin vermillion of the lips (*n* = 10) and proptosis (*n* = 8), while other observed facial features were a narrow nose (*n* = 5), narrow mouth (*n* = 3) and high palate (*n* = 2). Figure 4 and Supplementary Table S1.

### Cardiovascular

Arterial events (including aneurysms/dissections of the abdominal aorta, carotid artery and cerebral artery) have been previously reported in 10/150 (6.7%) molecularly confirmed pEDS patients (Kapferer-Seebacher et al., 2016; Kapferer-Seebacher et al., 2017; El Chehadeh et al., 2021). 10/21 individuals in this cohort underwent cardiac imaging and none were found to have any structural abnormalities of the heart. In 2/6 (33%) individuals who underwent arterial imaging, aneurysms were seen: in P18 a cerebral aneurysm (*n* = 1) in the form of a small brain aneurysm projecting posteriorly from the left internal carotid artery/anterior cerebral artery junction and in P19 a right internal carotid aneurysm (*n* = 1). Both individuals had leukoencephalopathy, and the aneurysms were detected in their early 30 s. In P16, normal imaging was reported after a transient ischaemic attack age 74. Spontaneous subarachnoid haemorrhage was the cause of death in P12 at age 30, although the underlying cause of the bleed is unclear. Intracranial haemorrhages have been previously reported in the literature (Kapferer-Seebacher et al., 2021). The risk of arterial events in individuals with pEDS remains unknown. Consideration of regular arterial surveillance is advised and has been reported to be conducted with a frequency of 1.5–2 years in some services (Kapferer-Seebacher et al., 2021).

### Central nervous system

White matter abnormalities have so far been detected in all patients with molecularly confirmed pEDS who have undergone cerebral imaging (10 of 10 reported patients, age range 8–68 years) (Kapferer-Seebacher et al., 2016; Kapferer-Seebacher et al., 2017; El Chehadeh et al., 2021). In this cohort, the majority of individuals (*n* = 12) did not have brain imaging at the time of reporting. In those that had imaging (*n* = 9), leukodystrophy was found in 8/9 (89%). P17, age 30, had no evidence of leukodystrophy on imaging. Leukodystrophy was discovered on average at age 40 (30–64 years of age), however this is dependent on age at imaging; no patients had prior imaging in childhood for comparison. The number of individuals from this cohort with these changes is likely to be underestimated as many have not undergone CNS imaging. There is currently no definite incidence data for leukodystrophy in the general adult population.

Leukodystrophies and leukoencephalopathies are a heterogenous group of disorders linked by white matter changes on imaging of the CNS which have recently been classified in 2015 (Vanderver et al., 2015). There are many distinct genetic leukoencephalopathies, and previous papers draw radiographic similarities between pEDS leukoencephalitis and genetic cerebral arteriopathies such as CADASIL (Cerebral Autosomal Dominant Arteriopathy with Subcortical Infarcts and Leukoencephalopathy) (Spranger et al., 1996; Kapferer-Seebacher et al., 2019). In contrast to these disorders and despite the radiographic similarities, neurological features are not severe, and in this cohort are mild or absent, with no evidence of cognitive decline. Neurological symptoms were reported by 11/21 (52%) individuals, including headaches (*n* = 8, 6 of which were migrainous), tremor (*n* = 3), partial bilateral palsy of fourth cranial nerve causing diplopia (*n* = 1), neuropathic pain (*n* = 2), poor memory (*n* = 4) and loss of concentration (*n* = 5). There was no evidence of lower motor neuropathology or speech disturbance. Vasculitis has previously been associated with a clinical diagnosis of pEDS (prior to available genetic testing) in an individual with periodontitis, osteolysis, cutaneous vasculitis and T cell reactivity to type 1 collagen (Hoffman et al., 1991). However, there are no currently available samples of cerebral tissue for further investigation of pathogenic mechanisms in this group.

Mental health disorder symptoms were reported in 9 individuals including alcohol dependence (*n* = 1), mixed anxiety and depression (*n* = 2), anxiety alone (*n* = 3) and depression alone (*n* = 3) associated with psychosis and a suicide attempt in 1 individual.

Currently there is no evidence that the leukodystrophy observed on imaging is linked to any specific degenerative or mental health disorder, and therefore cerebral imaging to monitor any leukodystrophy is not indicated unless the individual presents with neurological symptoms requiring imaging.

### Ear, nose, and throat

Vocal changes were noted in 8/21 (38%) individuals, in 3 individuals with a *C1R* VUS reported in Supplementary Appendix and have previously been reported in two individuals in the literature (George et al., 2016; Kapferer-Seebacher et al., 2019). Vocal changes have been previously reported in pEDS, and P13 has been reported before (George et al., 2016).

Vocal changes were defined as a hoarse voice often with associated high pitch. Onset of vocal changes was reported from early teens to 55 years (average age 27 years). Various causes for the vocal changes were reported: P2 developed subglottic stenosis with osseous metaplasia of the tracheal rings with vocal cord intermission and regurgitation, requiring tracheal resection which was complicated by an anastomotic stricture and required laser ablation, P13 developed subglottic stenosis below the larynx with an associated abnormality of the cricoarytenoid joint requiring surgical correction, in P14 recurrent laryngitis was reported with as many as 5 episodes a year, in P18 vocal cord sulci responded partially to speech and language therapy, and P21 developed tracheal stenosis which was treated surgically but required recurrent resections for significant scarring and has ongoing management with local steroid injections.

Subglottic stenosis is a fibrotic narrowing of the subglottic space which can occur in different situations including trauma, infection and systemic diseases. Previous papers have investigated an autoimmune hypothesis of acquired subglottic stenosis after finding autoantibodies to type II collagen in affected children, however, further investigations have not been conclusive (Stolovitzky and Todd, 1990; Stolovitzky et al., 1997). Autoantibodies to type II, IX and XI collagen have been found in relapsing polychondritis (RP), an autoimmune disease resulting in inflammation of cartilaginous structures and other organ systems including heart valves, eyes and vasculature (Borgia et al., 2018). 50% of these individuals develop laryngotracheobronchial involvement such as laryngomalacia and stenosis. The pathogenesis of RP is not fully understood, but animal models have shown that immune sensitization to ECM proteins can result in a clinical picture similar to RP (Loehrl and Smith, 2001).

The association between other vocal cord disorders and inflammatory and granulomatous diseases has been well documented, for example, fibro-inflammatory changes of the vocal cord commissure developing in rheumatoid arthritis (Eddaoudi et al., 2019).

### Molecular diagnosis

Both we and others have reported heterozygous missense or in-frame deletion variants within the *C1R* or *C1S* genes in individuals with pEDS. The majority of variants were located in *C1R* and affected the CUB2 and CCP1 domains (Figure 1) (Kapferer-Seebacher et al., 2016) and were found to have lost the ability to interact with C1q, preventing binding but otherwise forming and being secreted normally (Gröbner et al., 2019). However, P13 was included despite being reported in previous publications (George et al., 2016; Kapferer-Seebacher et al., 2016; Gröbner et al., 2019) due to the unusual site of the pathogenic *C1R* variant c.869A>G, p.(Asp290Gly); this is the only reported pathogenic variant which is located in the C1q binding site of C1r, appearing to inhibit binding of the C1r-C1s tetramer to C1q (Gröbner et al., 2019). See Supplementary Appendix.

## Conclusion

Lack of attached gingiva has been observed as early as 4 years of age and is pathognomonic for pEDS (Kapferer-Seebacher et al., 2020; Lepperdinger et al., 2022). This is the first study reporting the spectrum of extraoral clinical features assessed by specialists in inherited connective tissue disease, as observed in a series of 21 individuals with clinically and molecularly proven pEDS.

In our series, in line with previous observations, more than 80% had pretibial plaques, whilst vocal cord/laryngeal (38%) and white matter abnormalities in those that had imaging (89%) were also frequently encountered. Contrastingly, clinical features reflected in the minor criteria for a diagnosis of pEDS, namely, marfanoid facial features, acrogeria, cutaneous venous prominence and susceptibility to infection (Malfait et al., 2017) were less common and may merit revision.

Regular surveillance of the arterial tree could be considered, given the reported frequency of arterial aneurysms in this cohort (2/6, 33%) and previously reported arterial events (10/150) (El Chehadeh et al., 2021; Kapferer-Seebacher et al., 2021). Symptoms indicating concomitant autoimmune disorders or an acute abdomen should be investigated thoroughly.

Whilst there is clear clinical evidence of potentially abnormal connective tissue in pEDS as judged by features of hernias, skin and vascular fragility, other significant clinical features such as laryngeal thickening, white matter abnormalities or even the pretibial plaques are unexplained by a primary connective tissue matrix defect. Here, additional inflammatory mechanisms may be responsible and require further elucidation.

## Data Availability

The data presented in the study are deposited in the Global Variome shared LOVD database repository, please see https://databases.lovd.nl/shared/genes/C1R under the following IDs: C1R_000038, C1R_000028, C1R_000027, C1R_000030, C1R_000029, C1R_000009, C1R_000031, C1R_000032, C1R_000033, C1R_000034, C1R_000036, and C1R_000037.
