# Supplementary material for: Non-oral manifestations in adults with a clinical and molecularly confirmed diagnosis of periodontal Ehlers-Danlos syndrome
Source: Front Genet. 2023 May 31;14:1136339. doi: 10.3389/fgene.2023.1136339 (PMC10264792; doi:10.3389/fgene.2023.1136339)
Supplement: Supplementary file 1 [file DataSheet1.PDF]

Supplementary Appendix

Appendix 1.

Current understanding of pathogenic mechanisms in periodontal Ehlers-Danlos Syndrome (pEDS)

Complement 1 (C1) is the initiating factor in the classical complement pathway; activation of this molecule results in a cascade of proteins forming opsonins, anaphylatoxins and cell lysis complexes as part of the immune response.(Dunkelberger and Song, 2010) C1 is a multi-molecular complex comprising the recognition molecule C1q and two C1r and two C1s molecules which form a catalytic tetramer ($C1r_2C1s_2$) in the presence of increased extracellular calcium.(Arlaud et al., 2002; Mortensen et al., 2017; Almitairi et al., 2018; Gröbner et al., 2019)

C1q, C1r and C1s are all secreted into the extracellular space.(Lubbers et al., 2017) Within the extracellular space C1q is able to bind the $C1r_2C1s_2$ tetramer where it is held within the collagen-like structural region of the hexamer. (Mortensen et al., 2017) The recognition region of C1q binds activators such as antigen-antibody complexes; this results in a conformational change in C1q, initiating auto-activation of C1r which cleaves C1s; activated C1s initiates the downstream complement cascade by activating factors C2 and C4.(Gröbner et al., 2019) The presence of C1q is required for C1s activation.(Bally et al., 2019; Gröbner et al., 2019)

Identified (likely) pathogenic variants in *C1R* or *C1S* are thought to be gain of function. (Gröbner et al., 2019) Studies in fibroblasts from individuals with pEDS and healthy controls have shown that (likely) pathogenic *C1R* or *C1S* variants result in fragmentation of C1r or C1s molecules respectively, with intracellular retention of the interaction regions and secretion of catalytic, serine protease region fragments. (Gröbner et al., 2019) These catalytic C1r fragments have been found to retain the ability to activate normal/wild type C1s independently of C1q and cause the resulting downstream activation of C4 in vitro. (Gröbner et al., 2019) Cell cultures showed an increase in activated C4 in affected cells, but serum samples from these patients did not show a significantly increased level of systemic C4, or further downstream complement activation. (Gröbner et al., 2019) Interestingly, pathogenic *C1R/C1S* variants were largely found to have lost the ability to interact with C1q, preventing binding but otherwise forming and being secreted normally.

References

Almitairi, J. O. M., Girija, U. V., Furze, C. M., Simpson-Gray, X., Badakshi, F., Marshall, J. E., et al. (2018). Structure of the C1r–C1s interaction of the C1 complex of complement activation. *Proc Natl Acad Sci U S A*. doi: 10.1073/pnas.1718709115.

Arlaud, G. J., Gaboriaud, C., Thielens, N. M., and Rossi, V. (2002). Structural biology of C1. in *Biochemical Society Transactions* doi: 10.1042/BST0301001.

Bally, I., Dalonneau, F., Chouquet, A., Gröbner, R., Amberger, A., Kapferer-Seebacher, I., et al. (2019). Two Different Missense C1S Mutations, Associated to Periodontal Ehlers-Danlos Syndrome, Lead to Identical Molecular Outcomes. *Front Immunol* 10. doi: 10.3389/fimmu.2019.02962.

Dunkelberger, J. R., and Song, W. C. (2010). Complement and its role in innate and adaptive immune responses. *Cell Res* 20, 34–50. doi: 10.1038/cr.2009.139.

Ellard, S., Baple, E., Callaway, A., Berry, I., Forrester, N., Turnbull, C., et al. (2020). ACGS Best Practice Guidelines for Variant Classification in Rare Disease 2020. *Association for Clinical Genomic Science*. Available at: https://www.acgs.uk.com/quality/best-practice-guidelines/#VariantGuidelines [Accessed July 23, 2021].

Gröbner, R., Kapferer-Seebacher, I., Amberger, A., Redolfi, R., Dalonneau, F., Björck, E., et al. (2019). C1R Mutations Trigger Constitutive Complement 1 Activation in Periodontal Ehlers-Danlos Syndrome. *Front Immunol* 10. doi: 10.3389/fimmu.2019.02537.

Lubbers, R., van Essen, M. F., van Kooten, C., and Trouw, L. A. (2017). Production of complement components by cells of the immune system. *Clin Exp Immunol*. doi: 10.1111/cei.12952.

Mortensen, S. A., Sander, B., Jensen, R. K., Pedersen, J. S., Golas, M. M., Jensenius, J. C., et al. (2017). Structure and activation of C1, the complex initiating the classical pathway of the complement cascade. *Proc Natl Acad Sci U S A*. doi: 10.1073/pnas.1616998114.

Richards, S., Aziz, N., Bale, S., Bick, D., Das, S., Gastier-Foster, J., et al. (2015). Standards and guidelines for the interpretation of sequence variants: A joint consensus recommendation of the American College of Medical Genetics and Genomics and the Association for Molecular Pathology. *Genetics in Medicine*. doi: 10.1038/gim.2015.30.

Appendix 2.

Two families with a clinical diagnosis of periodontal Ehlers-Danlos Syndrome

Family 1

Family 1 reports 4 generations of clinically diagnosed periodontal Ehlers- Danlos Syndrome. Three individuals from 1 family (a mother, her daughter and her niece) have undergone genetic testing.

Proband (P.I-1)

The proband was a 54 year old female at the time of genetic diagnosis. She was born premature. She was diagnosed with Rheumatoid Arthritis at approximately age 29. Her medical history is extensive including two small bowel ruptures requiring bowel resections and complicated by incisional hernias, urinary retention, two premature births, recurrent acute sinusitis requiring endoscopic sinus surgery, dysphonia without stridor and without evaluation under anaesthetic, multiple cutaneous Basal Cell Carcinomas requiring skin grafting, severe ulceration of the lower limbs with recurrent staphylococcal skin infections and failed skin grafting. She had a history of easy bruising which would last for an extended period of time, and on the lower limbs contributed to pretibial plaques. She had no history of headache or neurological issues. There was no known history of arterial aneurysms

On examination she had diffuse skin fragility involving the shins, hands, forearms and face. She had pretibial discolouration with induration and ulceration. The discoloration extends around the full circumference of the calf. There was atrophic scarring limited to the shins and prominent venous vasculature on the hands with acrogeria. She had no hypermobility central or distally. She had bilateral varicose veins.

Intraoral photographs were assessed by a periodontal specialist demonstrating generalized lack of attached gingiva which is pathognomonic for pEDS.

Daughter of proband (P.II-1)

The daughter of the proband was genetically diagnosed at age 24. Born at 34 weeks gestation she had no features of prematurity. As a child she had no airway or voice impairments with no history of airway trauma or intubation. During late adolescence she developed stridor with dysphonia and was diagnosed with subglottic and glottic stenosis. Clinically the presentation was similar to stenosis seen

in granulomatosis with polyangiitis but her vasculitic screen was unremarkable. Initially managed endoscopically with balloon dilation and steroid injections this progressed to severe airway compromise requiring laryngotracheal reconstruction. She also had recurrent chest and sinus infections, with nasal mucosal changes similar to those seen in her airway. Like her mother, she bruises easily with this lasting for an extended period of time, contributing to pretibial plaques on the lower limbs and wounds ulcerating and being slow to heal. There was no history of arterial aneurysms and no known history of headaches or neurological conditions. Recent fertility investigations have discovered endometriosis.

On examination there was generalised skin fragility particularly involving the legs and including the hands, feet and face. She had significant pretibial discolouration which involves the entire circumference of the calf and extends to below the knee. Atrophic scarring was observed over the arms, ribcage and knees. The skin of the hands, wrists and feet was thin and translucent resulting in visible vasculature. She had distal hypermobility and bilateral knee joint pain.

Niece of proband (P.II-2)

The niece of proband one (daughter of the probands sister) was seen at age 19. As a child she had no airway or voice impairments with no history of trauma to the airway or intubation. During early adolescence she developed dysphonia and stridor. Airway assessment confirmed infraglottic stenosis requiring balloon dilatation every 12 weeks. Vasculitic and autoimmune screening was negative. More recently her disease has stabilized, with fixation of her cricoarytenoid joints resulting in dysphonia but no airway compromise. She has a history of easy but not prolonged bruising. Scoliosis had been diagnosed at the age of 14 years.

On examination she had a Beighton score of 2 with hypermobility in the fingers and wrists. She had skin fragility and pretibial discoloration and atrophic scarring.

Molecular analysis

A familial variant was identified in the proband and subsequently in all three individuals in *C1R* c.277G>T, p.Gly93Cys, affecting the CUB1 domain of C1r. However, on analysis the variant had been observed with an allele frequency of 0.06% (1:1600) in a European control population. Functional studies (Gröbner et al., 2019) found that this variant resulted in reduced secretion of C1r molecules

into the extracellular space. According to the ACMG classification, this variant currently remains a variant of uncertain significance (VUS)(Richards et al., 2015; Ellard et al., 2020). This is unsatisfactory given the clinical diagnosis of pEDS with lack of attached gingiva which appears pathognomonic for pEDS. However, the relatively high allele frequency of the variant within the control population cannot be ignored.

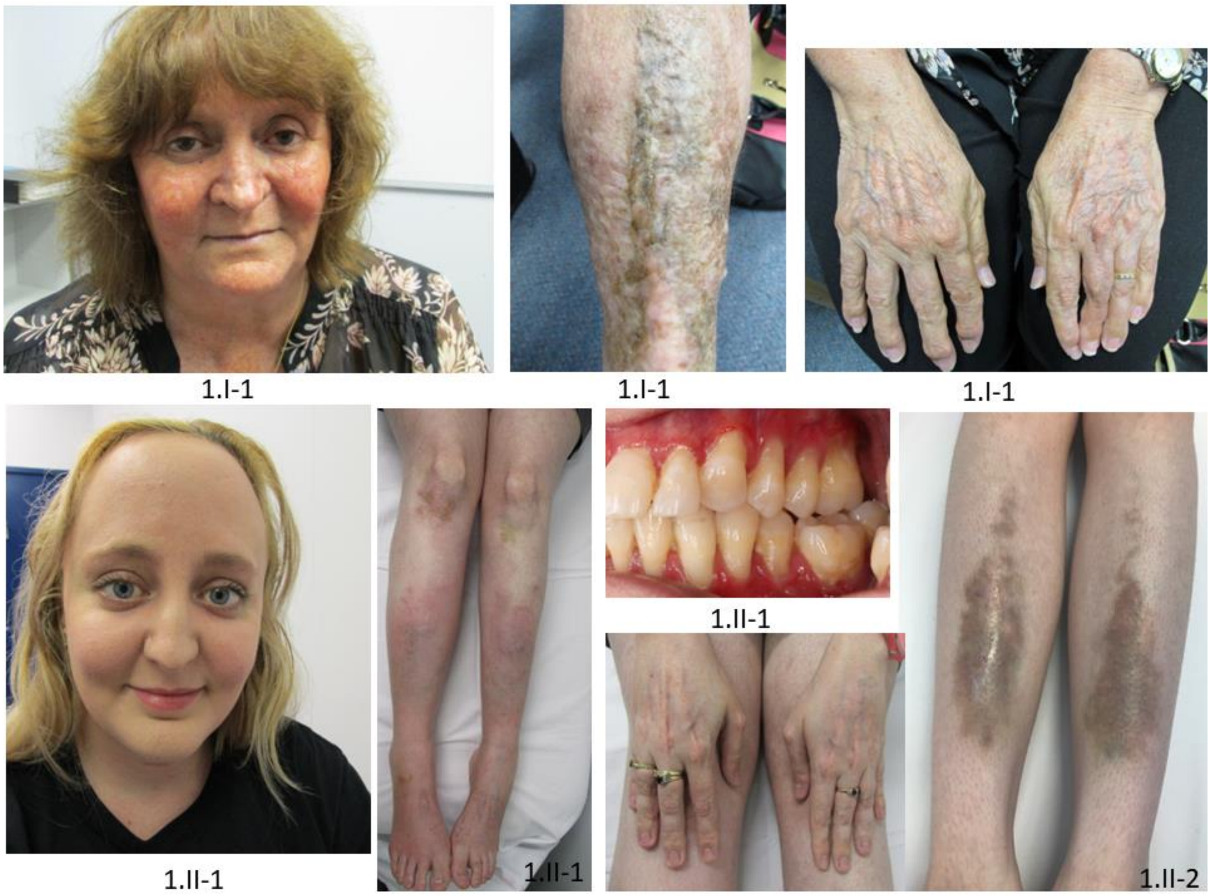

Family 2

Proband

The proband in this family was clinically diagnosed with pEDS at the age of 24 and was reassessed with genetic analysis at the age of 54. At birth she had no complications and met all her developmental milestones appropriately. She always bruised easily and was noted to have started to develop red discolouration of the shins at age 11 following trauma, which continued to progress and darken with age (see figure below). She was offered plastic surgery to treat the discolouration and underwent three procedures without success. She was diagnosed with lymphoedema in her early 20s with three episodes of cellulitis associated with this, and now which is managed with pressure stockings. She has had two children at the age of 24 and 38, both via C-section without known complications.

She has had a self-limiting three month episode of trigeminal neuralgia aged 51. Neurological imaging of the brain for investigation of chronic headaches identified diffuse white matter changes with no clear underlying cause. She has also been diagnosed with hypertension and type 2 diabetes mellitus and has required surgical intervention for dacryocystitis.

Recession of the gums was noted from the age of 30 onwards, but without a formal diagnosis of periodontitis. She has had some dental caries requiring fillings in childhood, and impacted molars removed in adulthood. A periodontal assessment for generalised lack of attached gingiva is pending.

On examination there was proptosis, in keeping with facial characteristics reminiscent of vascular EDS (vEDS). Some petechiae were noted on ear, hands and feet. She had a Beighton score of 3/9 with particular knee and back hypermobility. There were symmetrical darkly pigmented plaques circumferentially surrounding the shins and calves. Bilateral oedema of the ankles and bilateral piezogenic papules were also noted. The skin was not hyperextensible, and there were stretch marks over the abdomen. There was no evidence of spinal or thoracic deformity

Molecular analysis

The variant identified in this individual is a heterozygous variant of uncertain significance in *C1S* c.950T>A, p.Val317Glu, affecting the CCP1 domain of C1s (ACMG classification 3)(Richards et al.,

2015; Ellard et al., 2020). The variant is not currently listed in population-based variant databases and has not been previously reported in pEDS.

Family history

The proband is one of 7 siblings who have not yet undergone genetic assessment or investigation. Her mother was edentulous, however this happened later in life, and her father had lymphoedema in the lower limbs. Both parents are now deceased.

The proband has two children. Her son had experienced some discoloration on his shins after knocking his knees when playing football. He also had delayed eruption of some of his teeth and problems with dental overcrowding. The son underwent genetic testing for the identified variant of unknown significance (VUS) and was found to have the VUS as well. He was not assessed clinically as he sadly passed away due to a road traffic accident at the age of 18 years. The daughter is in her twenties. She is fit and well and does not have dental problems. She has not had genetic testing.

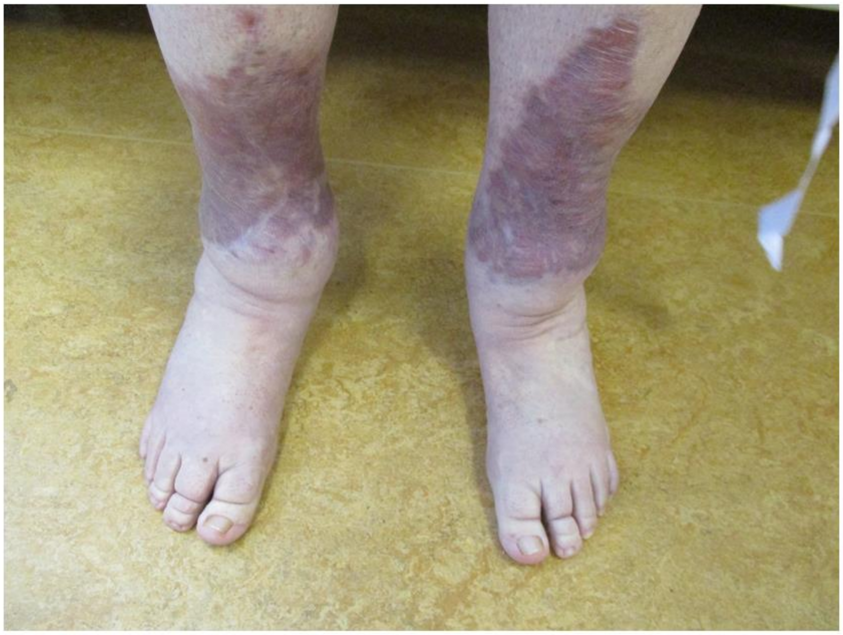

.

References

Ellard, S., Baple, E., Callaway, A., Berry, I., Forrester, N., Turnbull, C., et al. (2020). ACGS Best Practice Guidelines for Variant Classification in Rare Disease 2020. *Association for Clinical*

Genomic Science. Available at: https://www.acgs.uk.com/quality/best-practice-guidelines/#VariantGuidelines [Accessed July 23, 2021].

Richards, S., Aziz, N., Bale, S., Bick, D., Das, S., Gastier-Foster, J., et al. (2015). Standards and guidelines for the interpretation of sequence variants: A joint consensus recommendation of the American College of Medical Genetics and Genomics and the Association for Molecular Pathology. *Genetics in Medicine*. doi: 10.1038/gim.2015.30.
